# Genomic regions of speciation and adaptation among three species of grouse

**DOI:** 10.1038/s41598-018-36880-5

**Published:** 2019-01-28

**Authors:** Radoslav Kozma, Patrik Rödin-Mörch, Jacob Höglund

**Affiliations:** Department of Ecology and Genetics, Evolutionary Biology Centre, Uppsala University, Norbyvägen 18D, Uppsala, SE-75236 Sweden

## Abstract

Understanding the molecular basis of adaption is one of the central goals in evolutionary biology and when investigated across sister species it can provide detailed insight into the mechanisms of speciation. Here, we sequence the genomes of 34 individuals from three closely related grouse species in order to uncover the genomic architecture of speciation and the genes involved in adaptation. We identify 6 regions, containing 7 genes that show lineage specific signs of differential selection across the species. These genes are involved in a variety of cell processes ranging from stress response to neural, gut, olfactory and limb development. Genome wide neutrality test statistics reveal a strong signal of population expansion acting across the genomes. Additionally, we uncover a 3.5 Mb region on chromosome 20 that shows considerably lower levels of differentiation across the three grouse lineages, indicating possible action of uniform selection in this region.

## Introduction

The identification of genes involved in species’ adaptation to their environment is one of the main aims of evolutionary biology^1^. By uncovering the genetic basis of adaptation we improve our understanding of the mechanisms responsible for adaptation and speciation and by which phenotypic diversity arises. Until now, this was easier to achieve in domesticated animals where the strong artificial selection and constant, human-monitored environment quickly drove favoured alleles to fixation (e.g. chicken^2^ and horses^3^). This approach has proved fruitful in forming the foundations for the study of speciation. But in order to understand the way natural selection shapes individuals and species, study of wild populations has to be undertaken. This allows the examination of the architecture of adaptive traits under natural processes, free from intentional human-driven selection^4,5^.

One tool that enables the identification of genomic regions involved in speciation is the F_ST_ outlier method. By comparing the amount of differentiation at a particular locus to the overall levels of differentiation across the genome, the method indicates whether selection is acting upon any particular region. Higher levels of differentiation signal the presence of positive/directional selection, while lower levels of differentiation signal the presence of balancing/purifying selection. For example, the technique has been used to identify the gene controlling beak morphology in Darwin’s finches^6^, plumage colour in crows^7^ and adaptation to hypoxia in wolves^8^.

In this study, the aim is to utilise outlier methods to analyse the genomic architecture of species specific differences by exploring the genes involved in adaptation in three grouse; the willow grouse (*Lagopus lagopus lagopus*), the red grouse (*Lagopus lagopus scoticus*) and the rock ptarmigan (*Lagopus muta*). All belong to subfamily Tetraoninae and the *Lagopus* common ancestor diverged from the lineage leading to black grouse (*Tetrao tetrix*) and relatives approximately 3 million years ago (Mya)^9,10^. Consequently, the rock ptarmigan speciated from the *Lagopus lagopus* lineage around 2-1 Mya^9,10^. The red grouse is recognised as a subspecies of the willow grouse^11^, however it is endemic to the moorlands of Great Britain and Ireland and has thus been separated from continental populations at least since the time the British Isles separated from mainland Europe (~6000 years ago^12,13^). No record of any current gene flow between red grouse and willow grouse exists, with even the more proximal Irish and British red grouse showing no gene flow and substantial genetic differentiation^14^. Both the rock ptarmigan and the willow grouse have a circumpolar distribution, however, the rock ptarmigan occurs on Greenland, Iceland and Svalbard while the willow grouse does not^11,15^. Furthermore, the rock ptarmigan is the most cold adapted of the three, whereby it is a sedentary species that breeds across open arctic and subarctic habitats^16^. The willow grouse inhabits open subalpine habitat, boreal forests and moorland^11^. Where the distribution of the two species overlaps, the rock ptarmigan occurs at higher altitudes^17,18^. The three taxa also exhibit differences in plumage colouration; the rock ptarmigan and willow grouse have a brown summer plumage (full in females and partial in males) which moults into an all-white winter plumage, while both sexes of the red grouse forgo the white winter plumage instead remaining brown all year round^11^. The varying levels of arduous habitat and differences in plumage as well as population history thus provide a compelling system for the study of molecular adaptation and speciation.

## Results and Discussion

### Population structure and phylogeny

**Figure 1:**
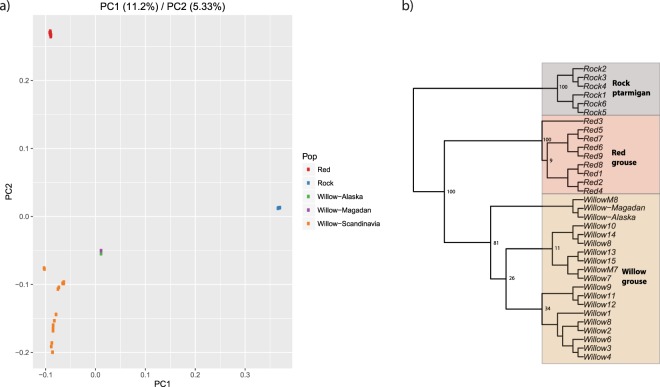
(**a**) Principal component analysis (PCA) and (**b**) Maximum likelihood inferred cladogram of the 34 grouse individuals used in the study (see Table S1 for sampling location of each individual). Bootstrap support values are given at each branch.

In total we identified 8 307 719 variable sites across the three taxa and a PCA based on these sites reveals a clear species clustering. PC1 separates the rock ptarmigan from the other two taxa (explaining 11.2% of the total variation) while PC2 further separates the willow and red grouse (explaining 5.33% of the total variation) (Fig. 1a). In the PCA, the willow grouse shows the most overall intraspecific variation, where the Scandinavian individuals show relatively large scatter while the Siberian and Alaskan individuals are more differentiated still (Fig. 1a). The red grouse and rock ptarmigan individuals show little intraspecific variation and cluster tightly together, but these differences in comparison to willow grouse most certainly is due to the relatively smaller and localised sample sizes for the latter two species. The species tree based on a random 59 574 autosomal SNP subset is in line with previously published phylogenies of the species complex^11,13,19^, whereby the red grouse is the sister taxon to the willow grouse (Fig. 1b). Intraspecific divergence largely followed expectations based on geographic origin.

### Genomic regions associated with differentiation

Overall levels of differentiation follow the prediction set by the phylogeny, with the lowest differentiation being between the willow grouse and red grouse (mean F_ST_ = 0.08 ± 0.04), followed by willow grouse and rock ptarmigan (mean F_ST_ = 0.17 ± 0.07) and the largest differentiation being between the red grouse and rock ptarmigan (mean F_ST_ = 0.21 ± 0.08, Supplementary Fig. 4).

Taxon specific test of neutrality revealed strong demographic effects acting across the whole genome in all three taxa. Tajima’s *D* was highly negative (genome-wide mean ± SD: willow grouse = −2.12 ± 0.13, red grouse = −2.31 ± 0.14, rock ptarmigan = −1.88 ± 0.09) while Fay and Wu’s *H* was positive (genome-wide mean ± SD: willow grouse = 0.32 ± 0.01, red grouse = 0.28 ± 0.01, rock ptarmigan = 0.37 ± 0.02, Supplementary Figs S5–S11), indicating strong signatures of population expansion in all three taxa.

**Figure 2:**
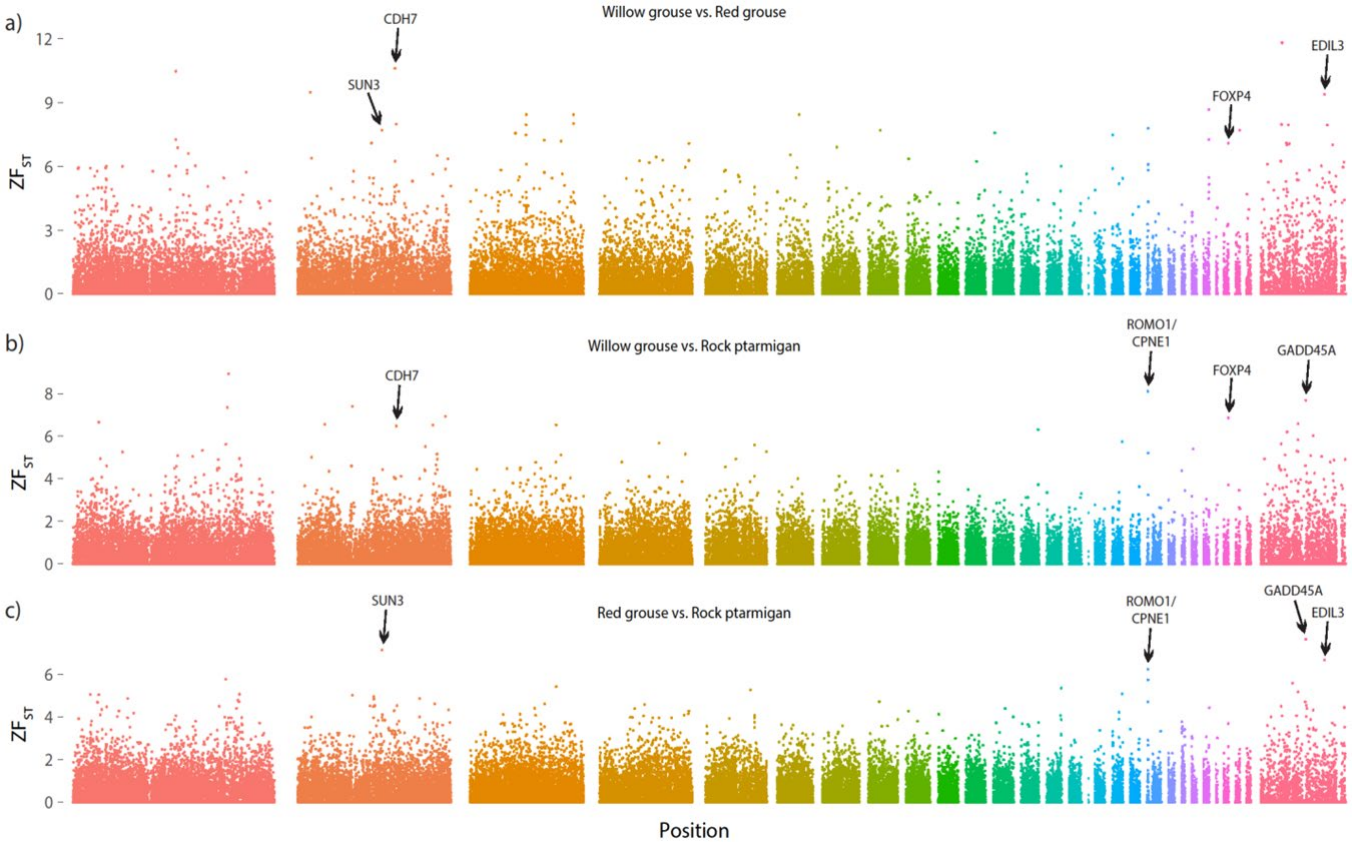
Genome-wide F_ST_ outlier test, showing the three pairwise comparisons of the three study taxa; (**a**) willow grouse vs. red grouse, (**b**) willow grouse vs. rock ptarmigan and c) red grouse vs. rock ptarmigan. The y-axis shows the ZF_ST_ score, where a 15 kb window with a score ≥6 is deemed an outlier. Each colour represents a different chromosome, with the autosomes arranged 1–28 (left to right) and chromosome Z located on the far right. The genes lying within same outlier window detected in two of the three comparisons are shown.

**Figure 3:**
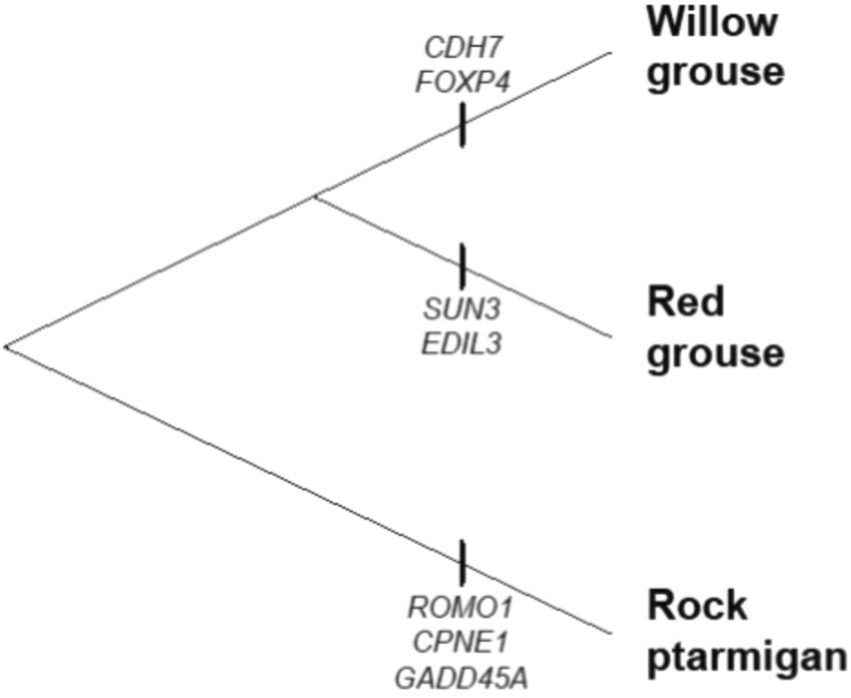
Genes under divergent selection in each of the three grouse lineages, as revealed by the F_ST_ outlier test.

The ZF_ST_ outlier test detected 2 non-overlapping regions for each taxon that were identified in more than one comparison (Table 1, Fig. 2). For the willow grouse these are located on chromosome 2 (position ~95 Mb) and 26; for the red grouse they are on chromosome 2 (position ~81 Mb) and Z (position ~61 Mb); for the rock ptarmigan they are on chromosome 20 and Z (position ~43 Mb). These regions include a number of genes as deduced from the alignment to the chicken genome - willow grouse: *CDH7* and *FOXP4;* red grouse: *SUN3* and *EDIL3*; rock ptarmigan: *ROMO1*, *CPNE1* and *GADD45A* (Fig. 3). Both *CDH7* and *FOXP4* are important developmental genes, with *FOXP4* being involved in neural, gut and pulmonary development and *CDH7* being integral in neural, optic, branchial and olfactory development, whose mutations in humans gives rise to the multiple-malformation ‘CHARGE’ syndrome^20,21^. *CPNE1* is part of the calcium-dependent membrane-binding protein family and plays a role in the neuronal progenitor cell differentiation^22^. *ROMO1* and *GADD45A* on the other hand are both involved in cellular stress response following reactive oxygen species (ROS) accumulation and environmental stress, respectively^23,24^. *EDIL3* (also known as *DEL-*1) is a gene involved in the cardiovascular system that has been shown to play a key function in angiogenesis in mice^25,26^. Lastly, *SUN3* is a gene involved in cytoskeletal anchoring and has been shown to play a role in mammal spermatogenesis^27^.

**Table 1:** Summary of the outlier windows detected across the three F_ST_ comparisons.

Species	Chromosome	Window (Mb)	Gene	Function
Willow grouse	2	95.610–95.625	*CDH7*	Cell adhesion protein involved in neural, optic, branchial and olfactory development
Willow grouse	26	4.800–4.815	*FOXP4*	Transcription factor involved in neural, gut and pulmonary development
Red grouse	2	81.855–81.870	*SUN3*	Protein anchor involved in cytoskeleton anchoring and spermatogenesis
Red grouse	Z	61.710–61.725	*EDIL3*	Integrin ligand involved in angiogenesis and vessel wall development
Rock ptarmigan	20	1.095–1.110	*ROMO1*	Involved in DNA damage and replication senescence by reactive oxygen species (ROS) build up
			*CPNE1*	Involved in calcium dependent neuronal cell differentiation
Rock ptarmigan	Z	43.650–43.665	*GADD45A*	Involved in cellular response to environmental stresses by activation of the p38/JNK pathway

*FOXP4*, which was found to be under positive selection in the willow grouse, is an interesting candidate gene for speciation. It is part of the forkhead box (FOX) group of transcription factors^28^, another member of which (*FOXP2*) has been shown to be highly involved in neural development, specifically important for learning of bird song and human speech^29,30^. Moreover, the product of the *FOXP4* gene has been shown to function in similar fashion to *FOXP2*^31^. This is interesting, because unlike in passerine birds, the technicality and repertoire of willow grouse calls are comparatively nominal. This raises two potential issues. Firstly, it is perhaps possible that the call repertoire of the willow grouse hides more intricacies than once thought and would thus select for higher call learning capability. Or, it may be the case that *FOXP4* has a more ubiquitous role in bird calls and may be selected for even in species without intricate song repertoires. Further complicating the picture, studies on the mouse have shown that the *FOXP4* transcription factor is also expressed in the developing pulmonary and gut tissue^32^, indicating the potential that this gene may also be the target of diet induced selection.

Even less is known about the specific function in birds of the remaining positively selected genes. *CDH7* appears to have a broad effect on early development, being involved in neural, optic, brachial and olfactory ontogeny^20,33^. Further studies in the chicken also reveal the gene’s important function in limb development, by regulation of limb bud mesenchymal cell motility and migration^34^. Due to such broad scope of possible traits in which *CDH7* can have an effect in the willow grouse it requires further research and insight to be able to specify more precisely what are the ramification of positive selection acting on this gene.

No experimental evidence exists for the function of *ROMO1* and *GADD45A* in birds; therefore it is hard to specify exactly why these genes are selected for in the rock ptarmigan. However, out of the three taxa studied here, the rock ptarmigan lives in the most extreme environment, so genes that would help cope with the effects of increased stress are likely to be more important for this species than for the other studied grouse. Likewise, no experimental evidence of *CPNE1*, *SUN3* or *EDIL3* in birds exists. Therefore, more will have to be known before we can hypothesise why any of the genes identified in this study might be positively selected in their respective grouse taxon. Furthermore, in all these regions of speciation, the identification of individual SNPs will aid in determining the overall target and effect of selection.

One methodological aspect of the F_ST_ outlier analysis is that it judges a window to be an outlier by looking at the number of standard deviations it is away from the overall mean. And since the range of F_ST_ values is bound between 0 and 1, the larger the overall mean, the harder it is for a window to be considered an outlier. As such, much fewer outliers were identified in the comparisons involving the rock ptarmigan, because the overall level of differentiation is higher (see mean F_ST_ values in Results and Supplementary Fig. S4). This was a conservative approach to eliminate capturing false positives. By lowering the threshold (e.g. consider anything with a ZF_ST_ >5 to be an outlier) might reveal more windows that are consistently identified as outliers among the different comparisons. However, different methods for identifying loci under selection consistently identify different loci even when applied to the same data^35,36^. To avoid false positives we felt a conservative cut-off was appropriate. This is also why we have chosen the same cut-off for all species contrasts.

### Region of low differentiation

**Figure 4:**
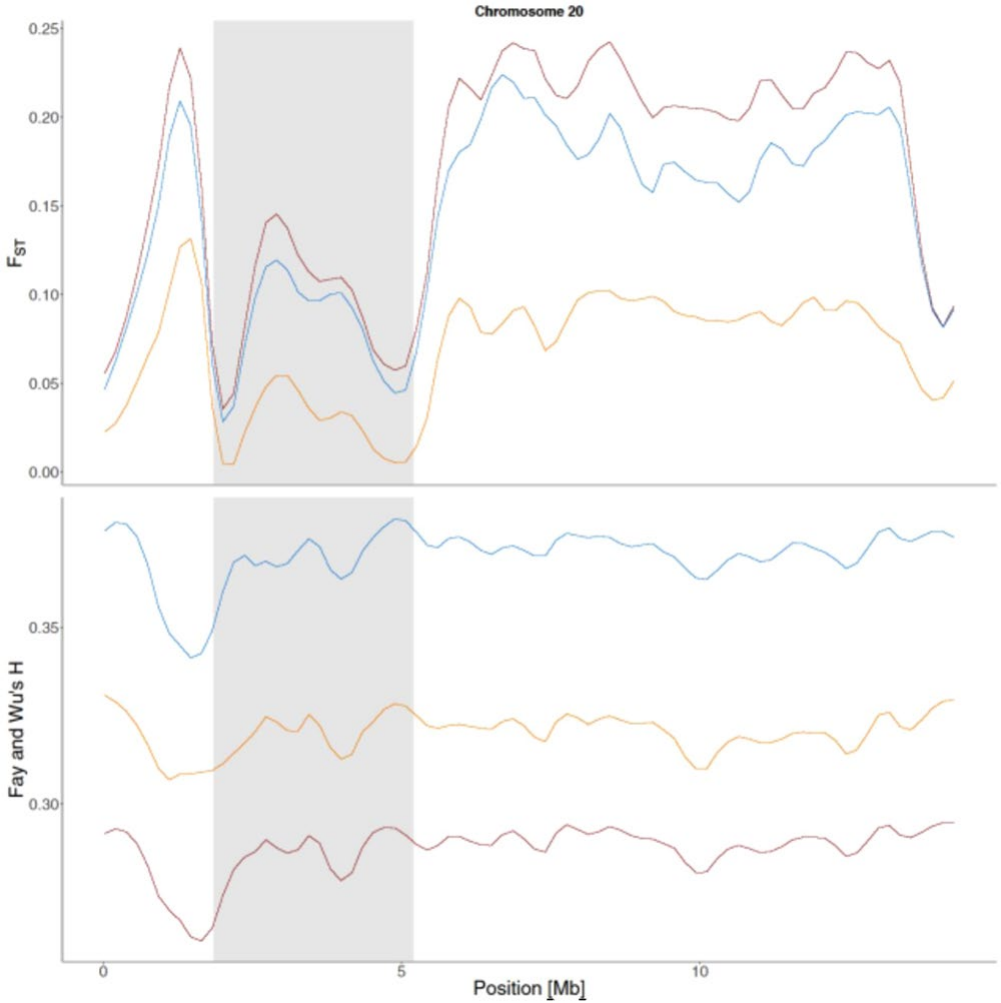
Chromosome 20, with the ~3.5-Mb long region of low differentiation highlighted in grey. Top panel shows the variation in F_ST_ (blue: willow grouse vs. rock ptarmigan, red: red grouse vs. rock ptarmigan, orange: willow grouse vs. red grouse), bottom panel shows the variation in Fay and Wu’s *H* (blue: rock ptarmigan, red: red grouse, orange: willow grouse).

Apart from finding regions of high differentiation, the F_ST_ outlier analysis also revealed a ~3.5 Mb region of low differentiation on chromosome 20, in which the F_ST_ values are considerably smaller when compared to the rest of the chromosome (Fig. 4). This pattern is seen in all three pairwise comparisons, suggesting the suppression of differentiation is acting across all three taxa. Blast alignment to the chicken genome revealed that coding sequences of 30 genes are present within the region (Supplementary Table S2). Interestingly, the first gene to overlap the start of this region (5′ end) is *ASIP*, also known as *Agouti*, which has a well-established role in melanogenesis^36–38^ but has also been shown to have a regulatory role in lipid metabolism in adipocytes^39^.

There are multiple scenarios that could account for this pattern. Recombination rates might be lower in this region, either, due to natural variation in recombination rate or suppressed due to genomic architecture like inversions. However, inversions lead to higher not lower signals of differentiation^40^. Uniform selection might be acting in the region, whereby the same alleles of all genes in this block are selected for in all three taxa. This would, in effect, produce the same long haplotype block in the three grouse. Alternatively, it could be that selection is acting on any of the genes within this region and the remainder of the block gets fixed through hitchhiking, but this should lead to reduced variation at linked sites.

The aforementioned strong demographic signal could, however, confound this inference. The significantly negative Tajima’s *D* and positive Fay and Wu’s *H* would indicate an overarching effect of population expansion and not selection shaping the genomic landscape of this region. However dips in Fay and Wu’s *H* statistic, while never reaching negative values, do signal that selection may also be acting.

From a functional perspective, the region contains the *Agouti* gene, which is situated immediately at its 5′ start. In mice studies, this gene has been shown to directly affect the coat colouration by binding to the *MC1R* cell surface protein thereby causing an increase in the production of pheomelanin (yellow/red pigment) and down-regulating the production of eumelanin (brown/black pigment)^37,41^. In quail it has also been shown that the *yellow* phenotype is caused by a mutation >90 kb upstream of the *Agouti* gene consequently changing its promoter^42^. In the three grouse species studied here, pigmentation plays an integral role in their ecology. Both the willow grouse and rock ptarmigan moult their brown summer plumage into a pure white plumage prior to winter in order to match the predominantly snowy environment. The red grouse on the other hand, living in the generally snow-free British Isles, forgoes this winter moult and instead retains its brown plumage year-round^43^. Because the genomic scan did not show any differentiation in this region between the red grouse and the other two species, this hints to the involvement of *Agouti* in the strongly conserved brown summer plumage, rather than the differentiated white-winter plumage. Further possible regulatory changes to *Agouti* or other genes involved in the melanogenesis pathway thus might be responsible for the change in winter plumage colour in the willow grouse and rock ptarmigan.

Complicating the issue further, the gene has also been found to be involved in regulation of lipid metabolism in mice and humans by antagonistically binding to another melanocortin receptor (*MC2R*)^39^. This raises the other possibility that the regulation of *Agouti* could be involved in the adaptation to the colder, more open habitats and sedentary lifestyle seen in all three grouse. However, more knowledge of the gene’s function in avian systems will have to be gathered before this issue can be fully resolved.

### Strong demographic signal

By performing tests of neutrality, we have also been able to show strong signals of expanding population across the whole genome of each of the studied taxa. This was revealed by a significantly negative Tajima’s *D* and a positive Fay and Wu’s *H*. This result goes in line with demographic studies on these species^44,45^, which show that the Scandinavian willow grouse (also containing the eventual red grouse lineage) and Greenland rock ptarmigan underwent a bottleneck during the Last Glacial Maximum (LGM) and only upon the deglaciation of northern Europe and Greenland did population sizes start to increase. Rock ptarmigan consistently showed higher genetic diversity based on θ than the other two taxa (Figs S5–S11).

A striking result in our data is the consistency of diversity patterns over chromosomal regions, seen in the supplementary figures (Figs S5–S11). The plots look almost identical for the three species, especially for the comparisons among red and willow grouse. Intuitively differential selection should cause more differences. A possible cause for this pattern could similar strong post/inter-glacial demographic effects^15,45^. A not mutually exclusive explanation may be consistent uniform purifying selection among these ecologically similar species which share a long common evolutionary history.

## Conclusions

Two avenues should be followed in order to understand the dynamics of grouse speciation better. The first is to explore the extent of recombination within the genomes. By studying the changes in recombination rate along chromosomes, further insight into the nature of selection acting upon particular regions can be gained^46,47^. If positive selection is indeed responsible for the outlier loci presented in this study, one would expect the suppression of recombination within the same and neighbouring outlier windows to follow.

The second necessary step is to further investigate the function of the genes discovered under selection in this study. Five out of the 7 genes found under divergent selection here have no experimental evidence of their function in avian systems, which makes the interpretation of the effect of selection acting on them difficult. To ultimately link gene function and adaptive phenotype, selection experiments should be carried out in order to test the consequences of different alleles^44^. Possible validation of these candidate genes can be achieved by a combination of selection experiments with resequencing and gene expression studies. Other, albeit less straightforward ways to validate function would applying functional assays such as knock-out/knock-down in the chicken at least, if not the grouse themselves.

Regardless this study provides an important genome-wide insight: grouse genomes vary among genomic regions as result of speciation and adaptation. Some parts are more divergent than expected by drift while other regions are more similar than expected by background differentiation. Regions which show signatures of positive and divergent selection vary among taxon comparisons while the region showing signs of uniform selection displays the same pattern across species contrasts.

## Methods

### Sampling, DNA extraction, sequencing and filtering

In total 34 individuals were sampled, comprising of 19 willow grouse (17 from Scandinavia, 1 from Magadan, Eastern Russia and 1 from Paxson, Alaska, USA), 9 red grouse from Yorkshire Dales National Park (Northern England) and 6 rock ptarmigan from south-western Greenland (Supplement Table S1). DNA extraction was performed using the Qiagen DNeasy Blood & Tissue Kit^®^ following the manufacturer’s instructions (Qiagen) and DNA quality of each individual was checked on an agarose gel and subsequently measured using a Quibit^®^ Fluorometer. After library preparation with the Illumina TruSeq protocol, the samples were sequenced at the SNP&SEQ technology platform of Uppsala University using an Illumina HiSeq. 2500 to generate 125 bp paired end reads with a target insert size of 350 bp. Quality trimming was performed using Trimmomatic v0.36^48^, following a 4 step procedure: (i) removing Illumina TruSeq adaptors, (ii) removing leading and trailing bases with quality score <5, (iii) scanning the read with a 4 base-pair sliding window and cutting when the average quality per base dropped below 15 and (iv) removing reads that were <50 bp after trimming. Overall read quality was checked using FastQC v0.11.4 (available at: http://www.bioinformatics.babraham.ac.uk/projects/fastqc).

### Mapping and Analysis

All properly paired reads that passed quality control were then mapped to the closely related black grouse genome^49^ using the BWA-MEM alignment algorithm^50^ with default setting. Duplicate reads were marked with Picard (http://broadinstitute.github.io/picard/) and local realignment around indels was performed with the GATK IndelRealigner tool^51,52^ producing the final filtered bam alignment files. The resultant mean coverage across all individuals was 28x (range: 23–38x, willow grouse mean: 29x, red grouse and rock ptarmigan mean: 28x) (Supplement Table S1, Supplement Figs S1–S3). All subsequent population genetic parameter estimation was based on genotype likelihoods obtained from these bam-alignment files.

We used the program ANGSD v0.902^53^ to calculate the unfolded allele frequency likelihoods for each taxon separately, using the black grouse reference genome to polarise the polymorphisms. The SAMtools genotyping model was implemented (-GL 1) and only sites that had mapping quality >50, base quality >30, Minor Allele Frequency (MAF) >0.05, minimum depth of [1/3 * mean taxon coverage * number of individuals] and maximum depth of [2 * mean taxon coverage * number of individuals] were considered. From the allele frequency likelihoods we then calculated the 2 dimensional site frequency spectrum (2D-SFS) for each taxon pair, which was then used to estimate the F_ST_ across non-overlapping 15 kb windows. The F_ST_ scores were subsequently Z-transformed and any region with a ZF_ST_ score above 6 was deemed an outlier^6^ and was subsequently aligned to the chicken genome (galGal4) using BLAST^54^ to identify gene content. The same outliers that were identified in both comparisons for each taxon (e.g. willow grouse vs. red grouse and willow grouse vs. rock ptarmigan) were thus assumed to be taxon specific and of higher confidence than outliers identified in one comparison but not the other.

From the allele frequency likelihoods we also estimated the unfolded marginal SFS for each taxon separately from which diversity and neutrality-test statistics (pairwise theta [π], Tajima’s *D*, Fay and Wu’s *H*^55^) were calculated across non-overlapping 15 kb windows. Principal component analysis (PCA) was performed using ngsTools^56^, whereby all individuals were genotyped together and subsequently a covariance matrix was created based on all variable sites.

To reconstruct the phylogeny of the taxa we first genotyped all individuals at a random subset of 500 k autosomal SNPs using ANGSD’s SAMtools genotyping model. In order to remove redundancy due to linkage disequilibrium (LD), we filtered the dataset using SNPhylo^57^ applying the LD threshold of 0.4 according to the authors recommendations for genomes with relatively high levels of LD, leaving 59 574 informative SNPs. Maximum likelihood phylogeny tree was subsequently inferred using the rapid bootstrapping algorithm with the “GTRGAMMA” substitution model and bootstrapping criterion set to “autoMRE” employed in RaxML v8.2.4^58^.

All methods were carried out in accordance with relevant guidelines and regulations and all experimental protocols followed Uppsala University guidelines. None of the protocols involved live animals and birds were hunted under the guides and regulations of each respective country where a bird was shot^59^.

## Supplementary information

Supplementary Information

## Data Availability

Raw data is archived at the sequence read archive (SRA) under bioproject acession number: PRJNA512999.
