# Supplementary material for: Genomic regions of speciation and adaptation among three species of grouse
Source: Sci Rep. 2019 Jan 28;9:812. doi: 10.1038/s41598-018-36880-5 (PMC6349846; doi:10.1038/s41598-018-36880-5)

Supplementary Information

Genomic regions of speciation and adaptation among three species of grouse

Radoslav Kozma, Patrik Rödin-Mörch, Jacob Höglund

Department of Ecology and Genetics, Evolutionary Biology Centre, Uppsala University, Norbyvägen 18D, Uppsala, SE-75236, Sweden

Supplement Table S1. Sample information.

**Sample**	**Species**	**Location**	**Tissue**	**Sample year**	**Mean coverage (x)**
Willow1	*L. lagopus lagopus*	Smøla, Norway	Muscle	2006	27.0
Willow2	*L. lagopus lagopus*	Frøya, Norway	Muscle	2006	38.4
Willow3	*L. lagopus lagopus*	Frøya, Norway	Muscle	2006	38.5
Willow4	*L. lagopus lagopus*	Frøya, Norway	Muscle	2006	30.8
Willow5	*L. lagopus lagopus*	Frøya, Norway	Muscle	2006	27.1
Willow6	*L. lagopus lagopus*	Frøya, Norway	Muscle	2006	33.3
Willow7	*L. lagopus lagopus*	Lierne, Norway	Spleen	2006	30.5
Willow8	*L. lagopus lagopus*	Dovre, Norway	Spleen	2005	27.3
Willow9	*L. lagopus lagopus*	Dovre, Norway	Spleen	2005	29.4
Willow10	*L. lagopus lagopus*	Dovre, Norway	Spleen	2005	26.1
Willow11	*L. lagopus lagopus*	Dovre, Norway	Spleen	2005	31.1
Willow12	*L. lagopus lagopus*	Dovre, Norway	Spleen	2005	28.0
Willow13	*L. lagopus lagopus*	Dovre, Norway	Spleen	2005	24.8
Willow14	*L. lagopus lagopus*	Dovre, Norway	Spleen	2005	28.8
Willow15	*L. lagopus lagopus*	Dovre, Norway	Spleen	2005	28.8
WillowM7	*L. lagopus lagopus*	Jämtland, Sweden	Muscle	1995	23.5
WillowM8	*L. lagopus lagopus*	Jämtland, Sweden	Muscle	1995	31.3
Willow-Alaska	*L. lagopus lagopus*	Paxson, Alaska, USA	Liver	1995	26.8
Willow-Magadan	*L. lagopus lagopus*	Magadan, Russia	Blood	1995	26.5
Red1	*L. lagopus scoticus*	Feetham, Yorkshire Dales, UK	Liver	2013	26.5
Red2	*L. lagopus scoticus*	Feetham, Yorkshire Dales, UK	Liver	2013	26.9
Red3	*L. lagopus scoticus*	Feetham, Yorkshire Dales, UK	Liver	2013	30.3
Red4	*L. lagopus scoticus*	Feetham, Yorkshire Dales, UK	Liver	2013	26.3
Red5	*L. lagopus scoticus*	Gunnerside, Yorkshire Dales, UK	Liver	2013	29.6
Red6	*L. lagopus scoticus*	Gunnerside, Yorkshire Dales, UK	Spleen	2013	26.8
Red7	*L. lagopus scoticus*	Gunnerside, Yorkshire Dales, UK	Spleen	2013	27.3
Red8	*L. lagopus scoticus*	Gunnerside, Yorkshire Dales, UK	Liver	2013	27.4
Red9	*L. lagopus scoticus*	Gunnerside, Yorkshire Dales, UK	Liver	2013	28.6
Rock1	*L. muta*	Qasigiannguit, Greenland	Muscle	2007	26.7
Rock2	*L. muta*	Qasigiannguit, Greenland	Muscle	2007	27.1
Rock3	*L. muta*	Qasigiannguit, Greenland	Muscle	2007	27.3
Rock4	*L. muta*	Qasigiannguit, Greenland	Muscle	2007	30.4

Rock5	*L. muta*	Qasigiannguit, Greenland	Muscle	2007	28.6
Rock6	*L. muta*	Qasigiannguit, Greenland	Muscle	2007	27.4

Supplement Table S2. Summary of the 30 genes found in the 3.5-Mb region of low differentiation on chromosome 20

Gene	Function
***ASIP* (*Agouti*)**	Causes hair follicle melanocytes to synthesize pheomelanin
AHCY	Involved in the control of methylation
RALY	Thought to play a role in pre-mRNA splicing and embryonic development
EIF2B	Involved in protein synthesis
CHMP4B	Functions in the sorting of endocytosed cell-surface receptors
ZNF341	May be involved in transcription regulation
CBFA2T2	Involved in the promotion of leukemogenesis
PXMP4	Involved in the peroxisome pathway
E2F1	Transcription factor involved in the cell cycle
NECAB3	Plays a role in beta-amyloid generation
TOX2	Transcription factor involved in chromatin binding
SNTA1	Encodes the most common syntrophin isoform found in cardiac tissues
PLIN3	Fatty acid, triacylglycerol, and ketone body metabolism
ACSS2	Lipid synthesis and energy production
EDEM2	Endoplasmic reticulum associated degradation of glycoproteins
TRPC4AP	Transport of glucose, other sugars, bile salts and organic acids
SOGA1	Involved in autophagy
MYH7B	Important for muscle contraction
GSS	Synthesis of glutathione
PTPRT	Signalling molecule that involved in cell growth, differentiation, mitotic cycle
ENSGALG00000003494	Novel predicted protein
SLC32A1	Integral membrane protein involved in gamma-aminobutyric acid (GABA) and glycine uptake into synaptic vesicles
IFT52	Involved in the assembly, maintenance and functioning of primary cilia
DHX35	Regulates cellular processes involving alteration of RNA secondary structure
FAM83D	Required for proper chromosome congression and alignment during mitosis
SGK2	Involved in the regulation of ion channels, membrane transporters, cell growth, survival and proliferation
ACTR5	Involved in transcriptional regulation and DNA replication
MYBL2	Involved in cell cycle progression
ARHGAP40	GTPase activator
PPP1R16B	Regulator of pulmonary endothelial cell (EC) barrier function

Figure S1. Quality information of the filtered willow grouse bam files: a) distribution of base quality scores, b) distribution of read depth across all 19 samples and c) distribution of read depth for each sample separately.

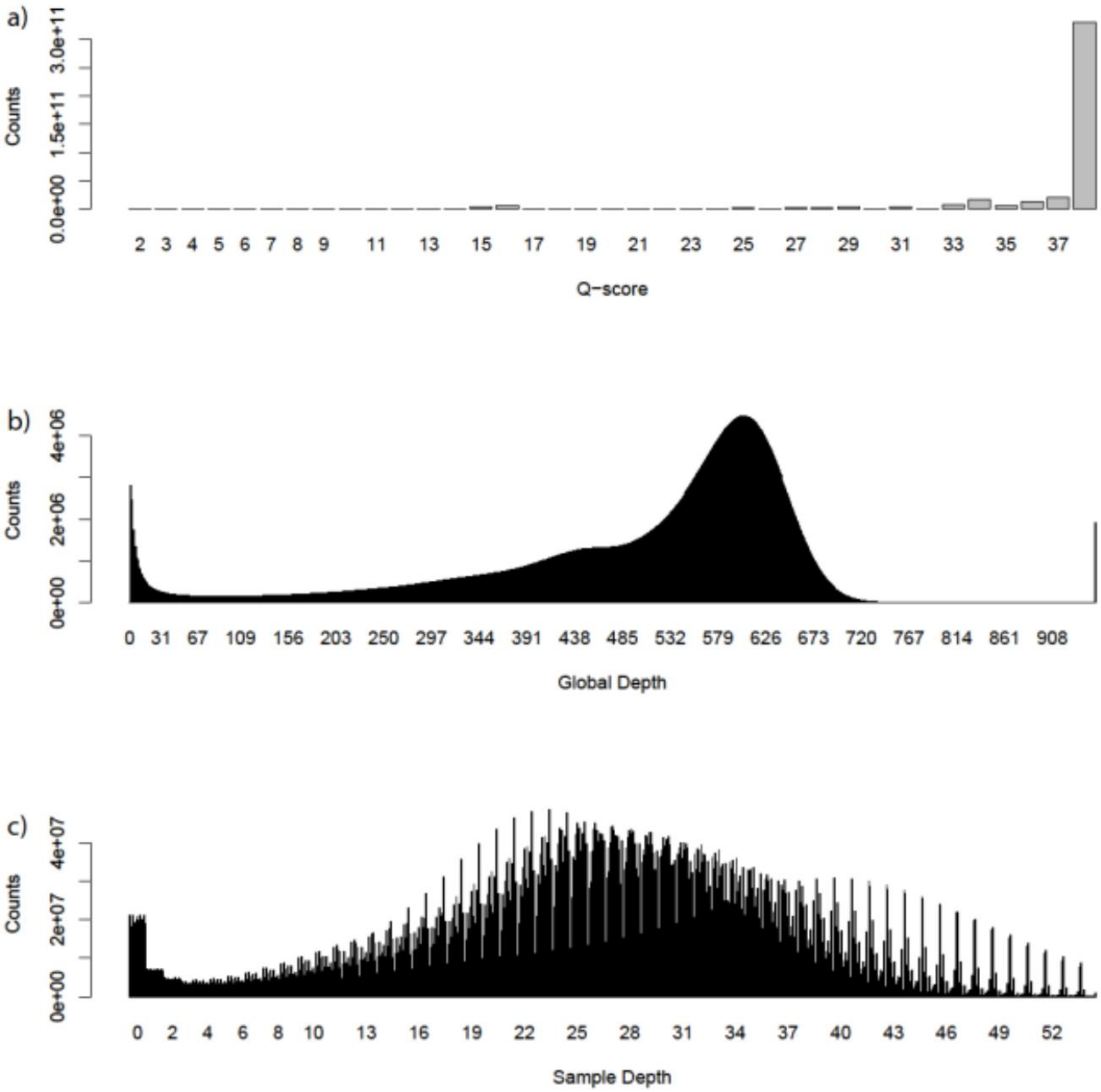

Figure S2. Quality information of the filtered red grouse bam files: a) distribution of base quality scores, b) distribution of read depth across all 19 samples and c) distribution of read depth for each sample separately.

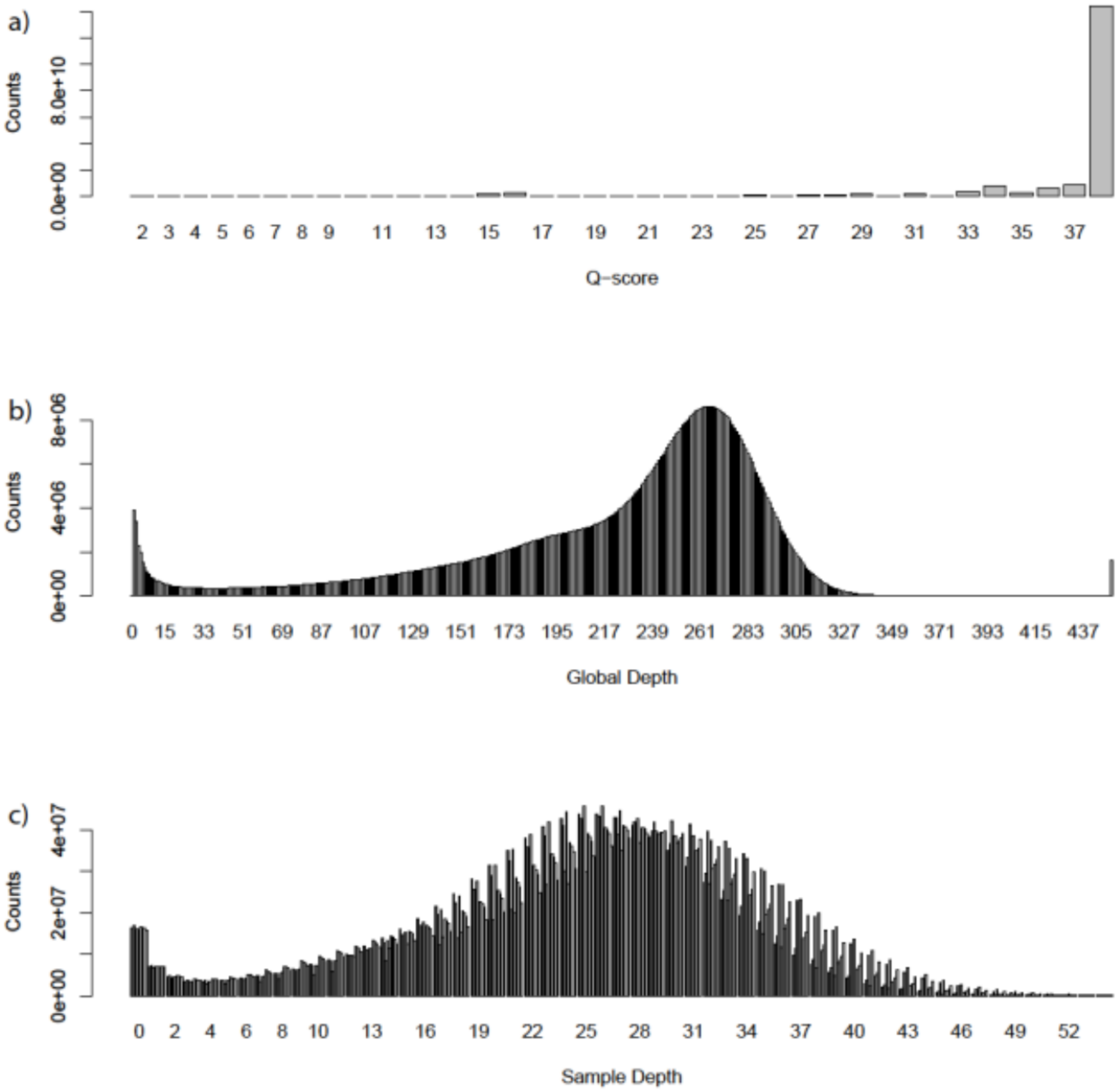

Figure S3. Quality information of the filtered rock ptarmigan bam files: a) distribution of base quality scores, b) distribution of read depth across all 19 samples and c) distribution of read depth for each sample separately.

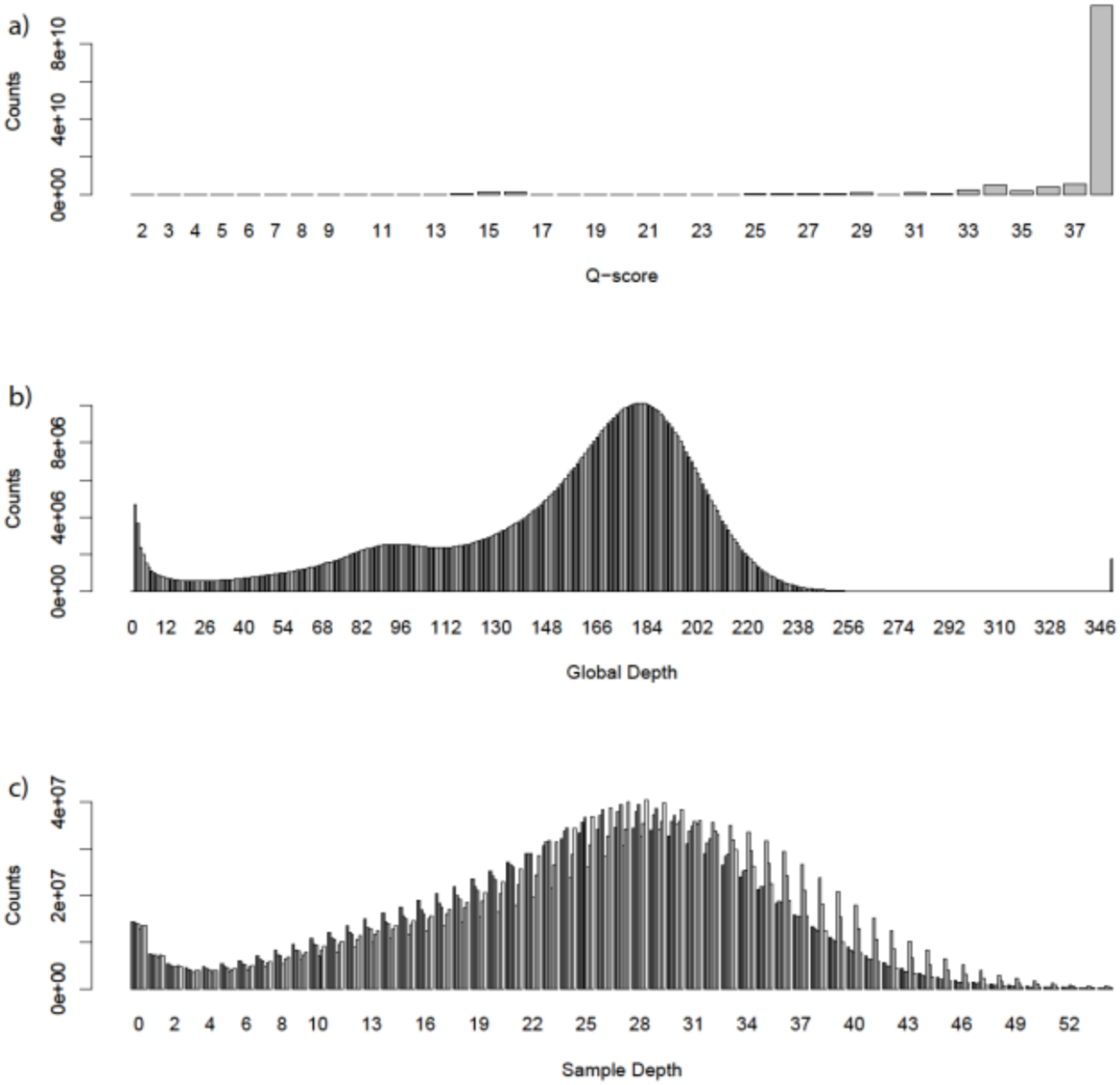

Figure S4. Violin plot showing the overall distribution of F_{ST} values across the three comparisons.

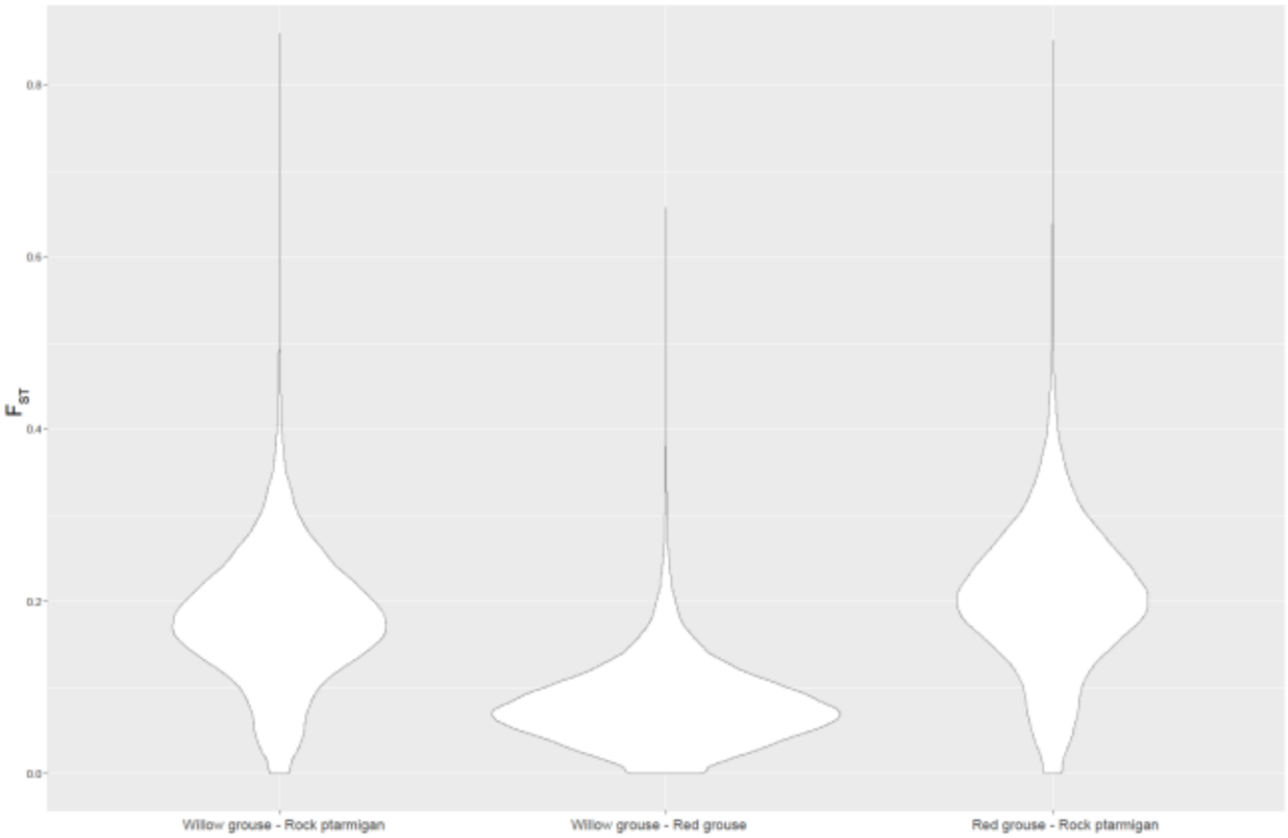

Figure S5. Variation in pairwise θ, Tajima's *D* and Fay and Wu's *H* across chromosomes 1-5 (running left to right) in the three studied taxa. Blue: rock ptarmigan, red: red grouse, orange: willow grouse.

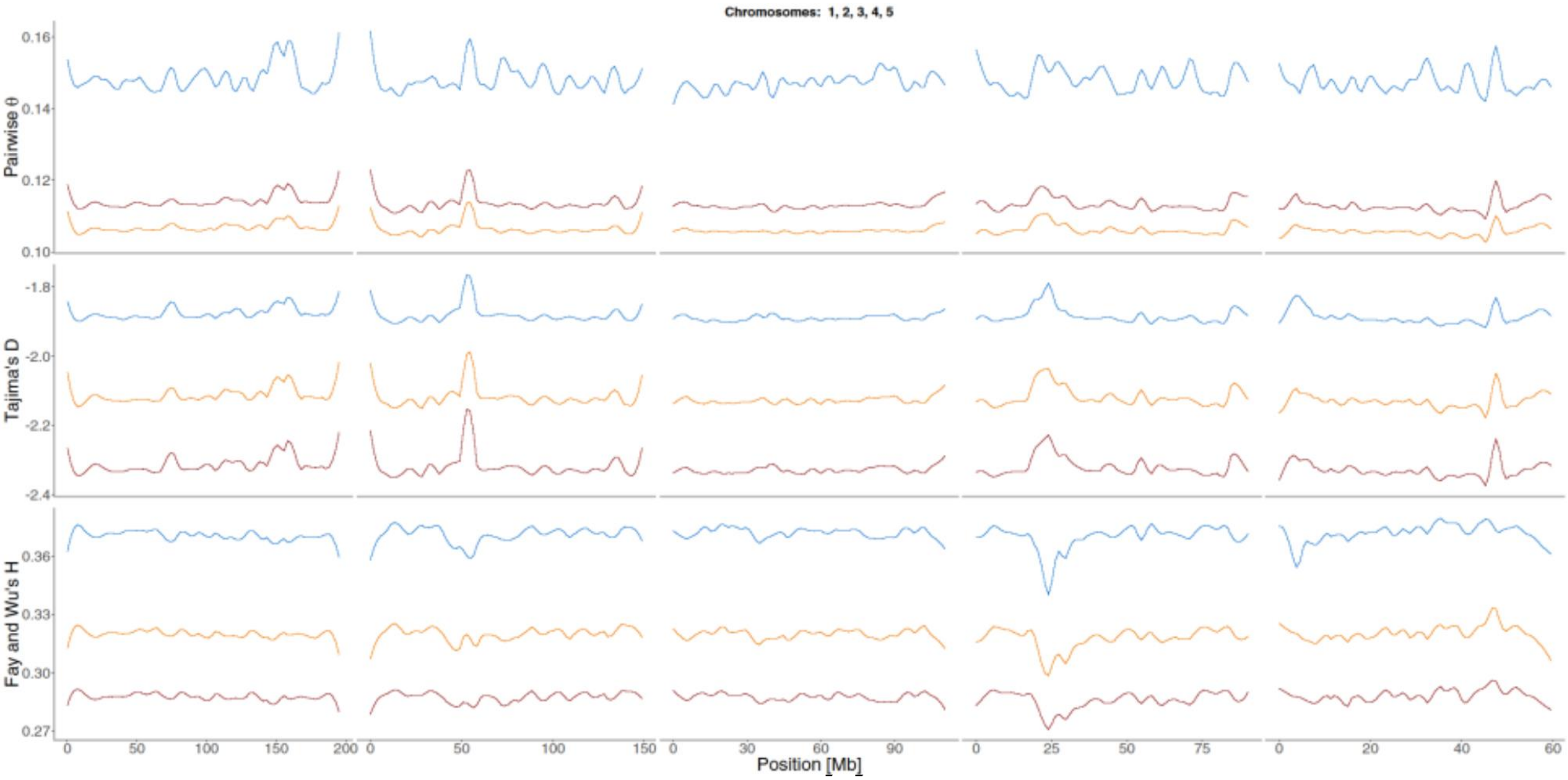

Figure S6. Variation in pairwise θ, Tajima's *D* and Fay and Wu's *H* across chromosomes 6-10 (running left to right) in the three studied taxa. Blue: rock ptarmigan, red: red grouse, orange: willow grouse.

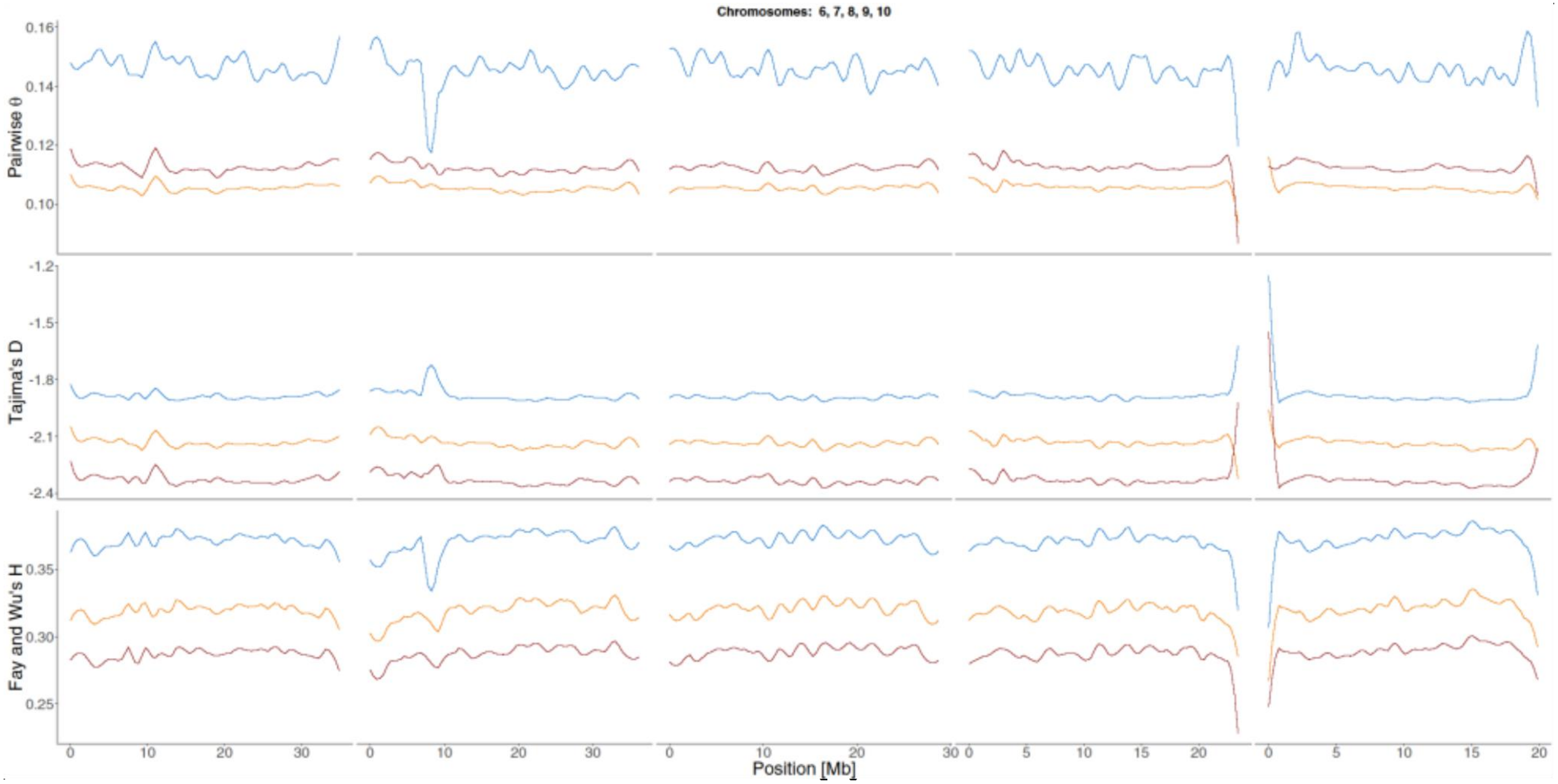

Figure S7. Variation in pairwise θ, Tajima's *D* and Fay and Wu's *H* across chromosomes 11-15 (running left to right) in the three studied tax. Blue: rock ptarmigan, red: red grouse, orange: willow grouse.

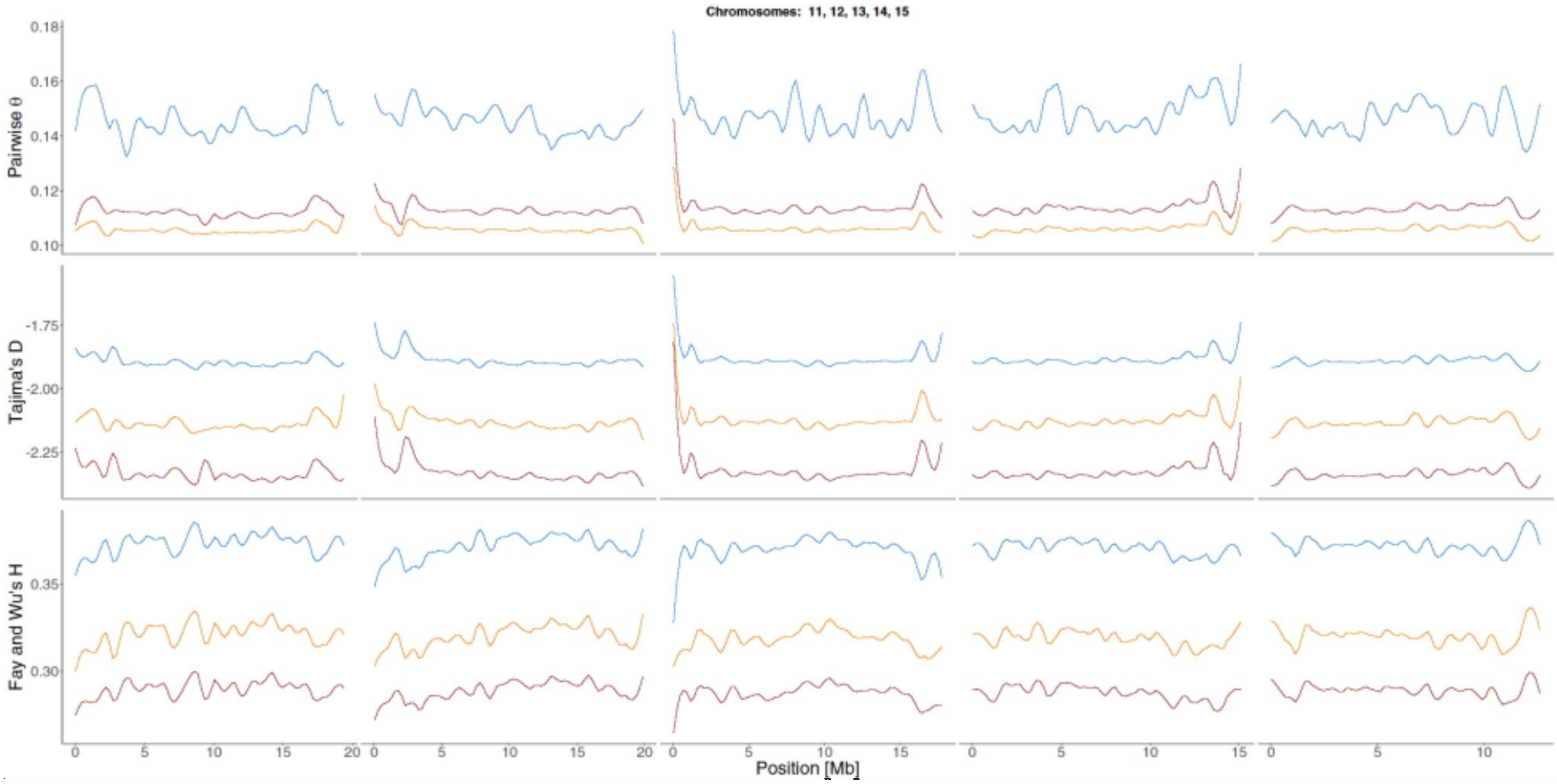

Figure S8. Variation in pairwise θ, Tajima's *D* and Fay and Wu's *H* across chromosome 16 (poorly mapped due to the highly variable MHC region) in the three studied taxa. Blue: rock ptarmigan, red: red grouse, orange: willow grouse.

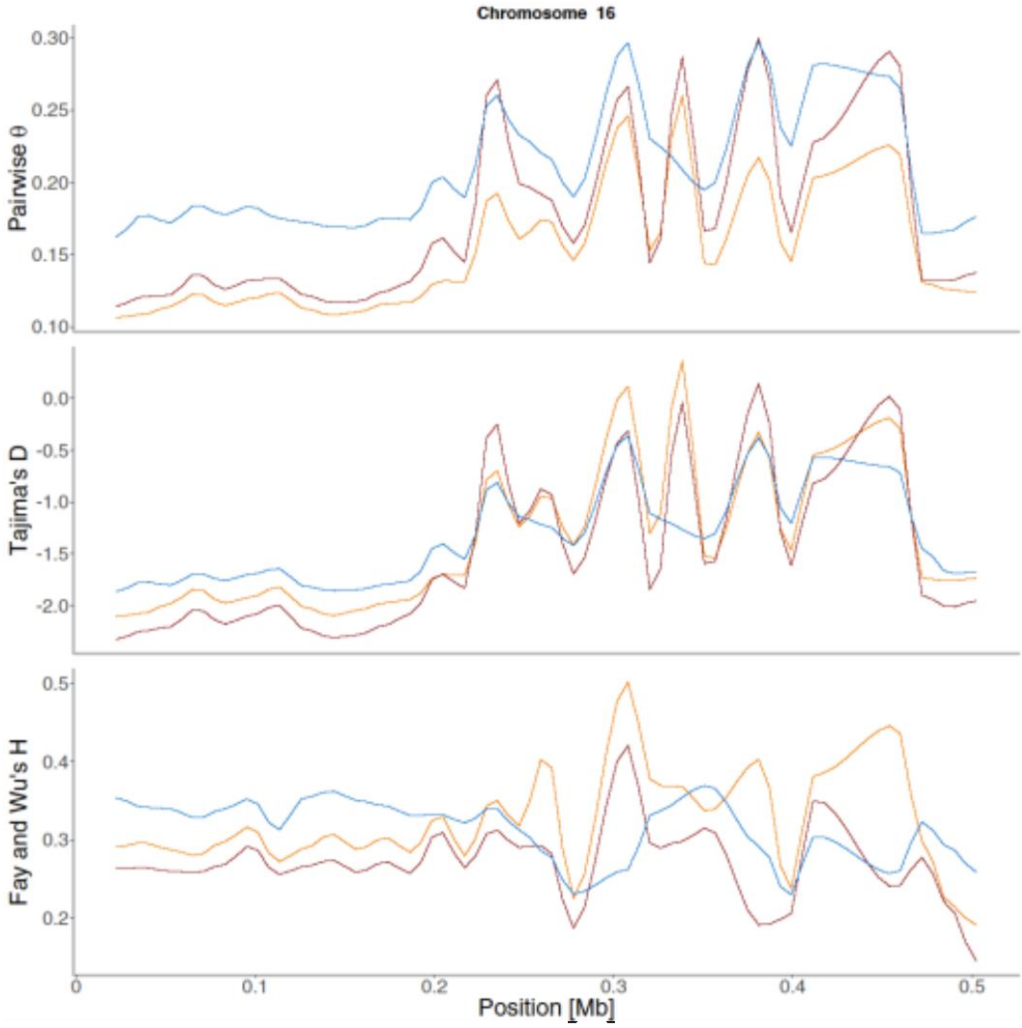

Figure S9. Variation in pairwise θ, Tajima's *D* and Fay and Wu's *H* across chromosomes 17-22 (running left to right) in the three studied taxa. Blue: rock ptarmigan, red: red grouse, orange: willow grouse.

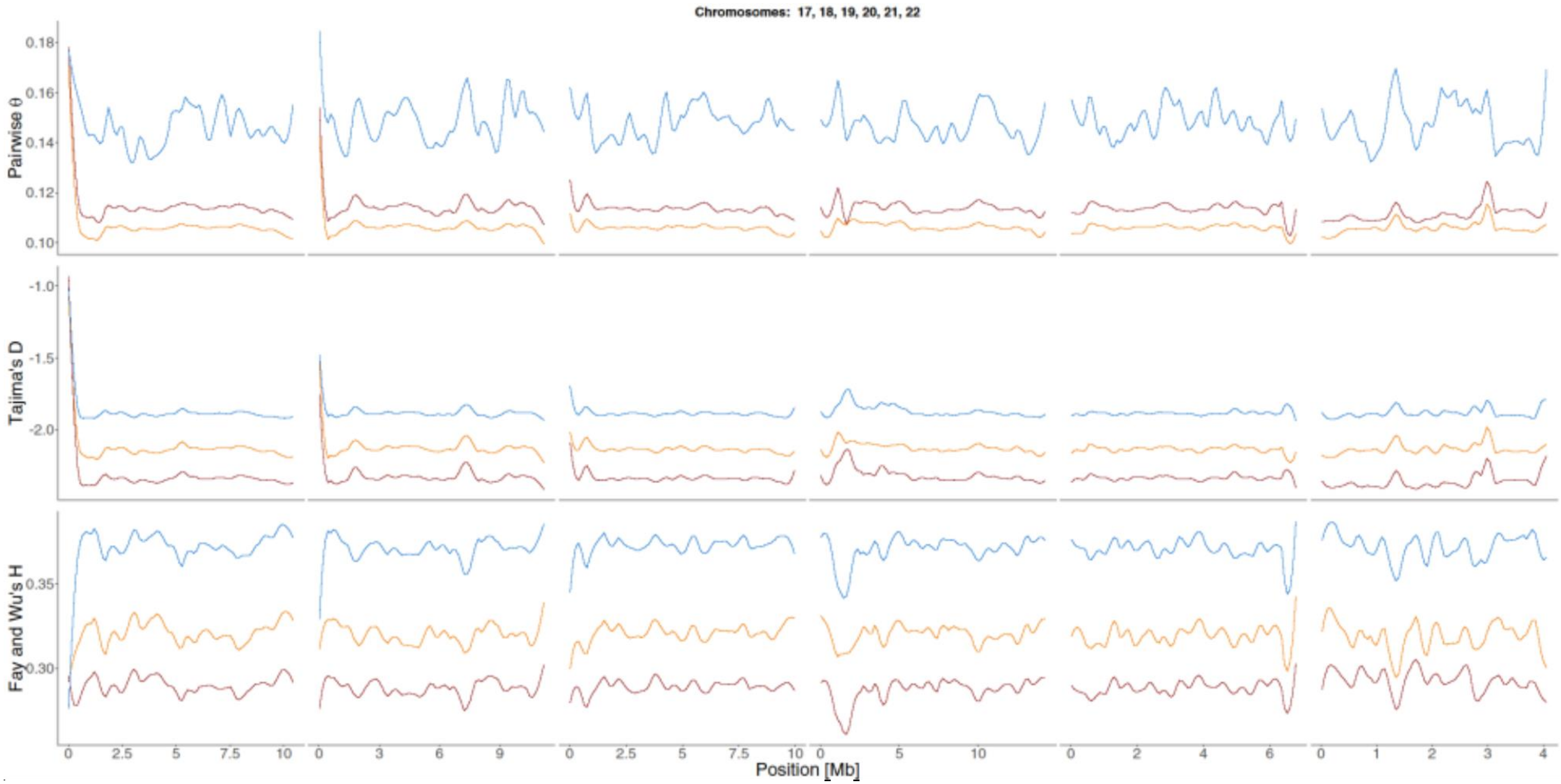

Figure S10. Variation in pairwise θ, Tajima's *D* and Fay and Wu's *H* across chromosomes 23-28 (running left to right) in the three studied taxa. Blue: rock ptarmigan, red: red grouse, orange: willow grouse.

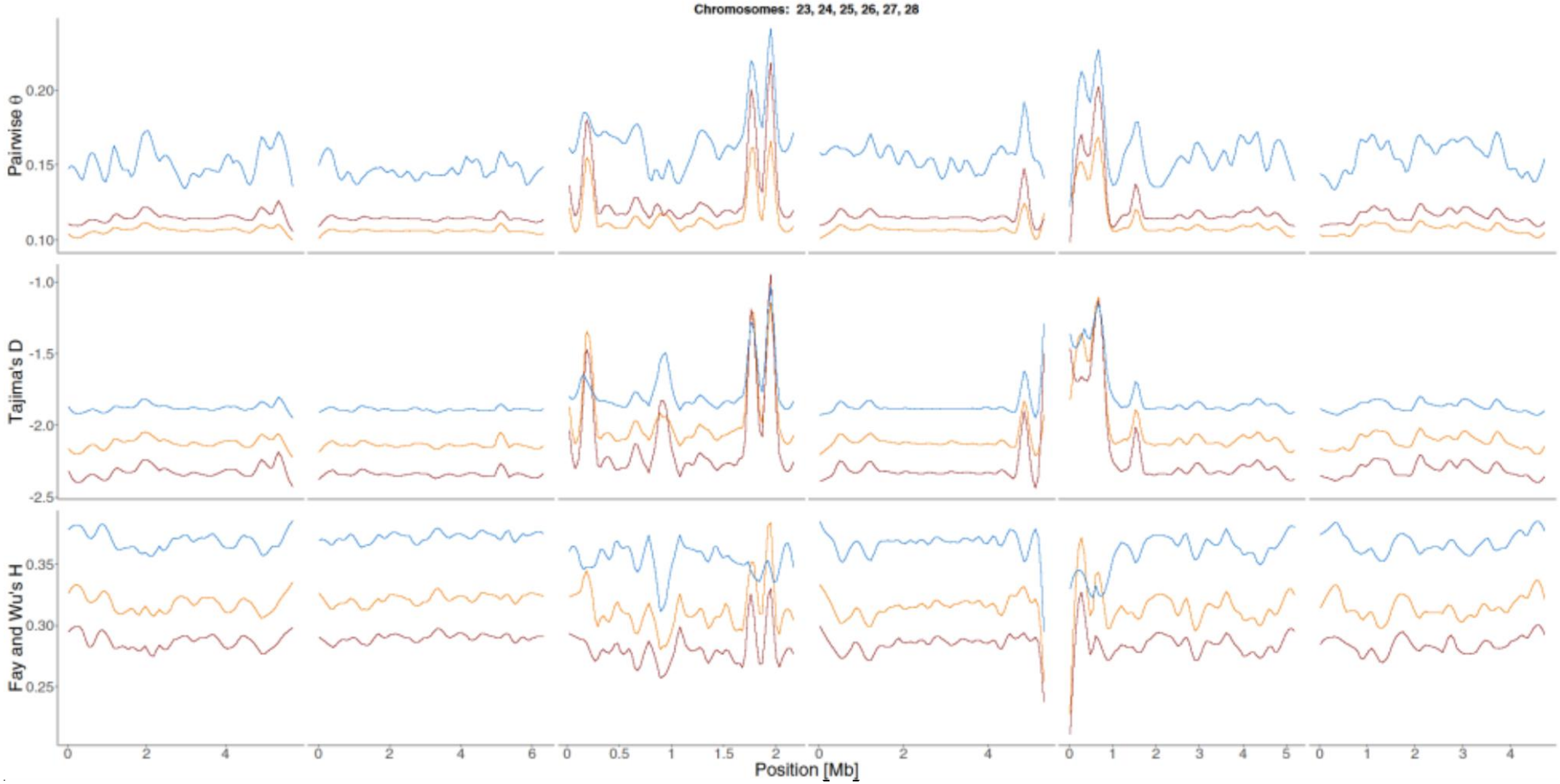

60

Figure S11. Variation in pairwise θ, Tajima's *D* and Fay and Wu's *H* across chromosome Z in the three studied taxa. Blue: rock ptarmigan, red: red grouse, orange: willow grouse.

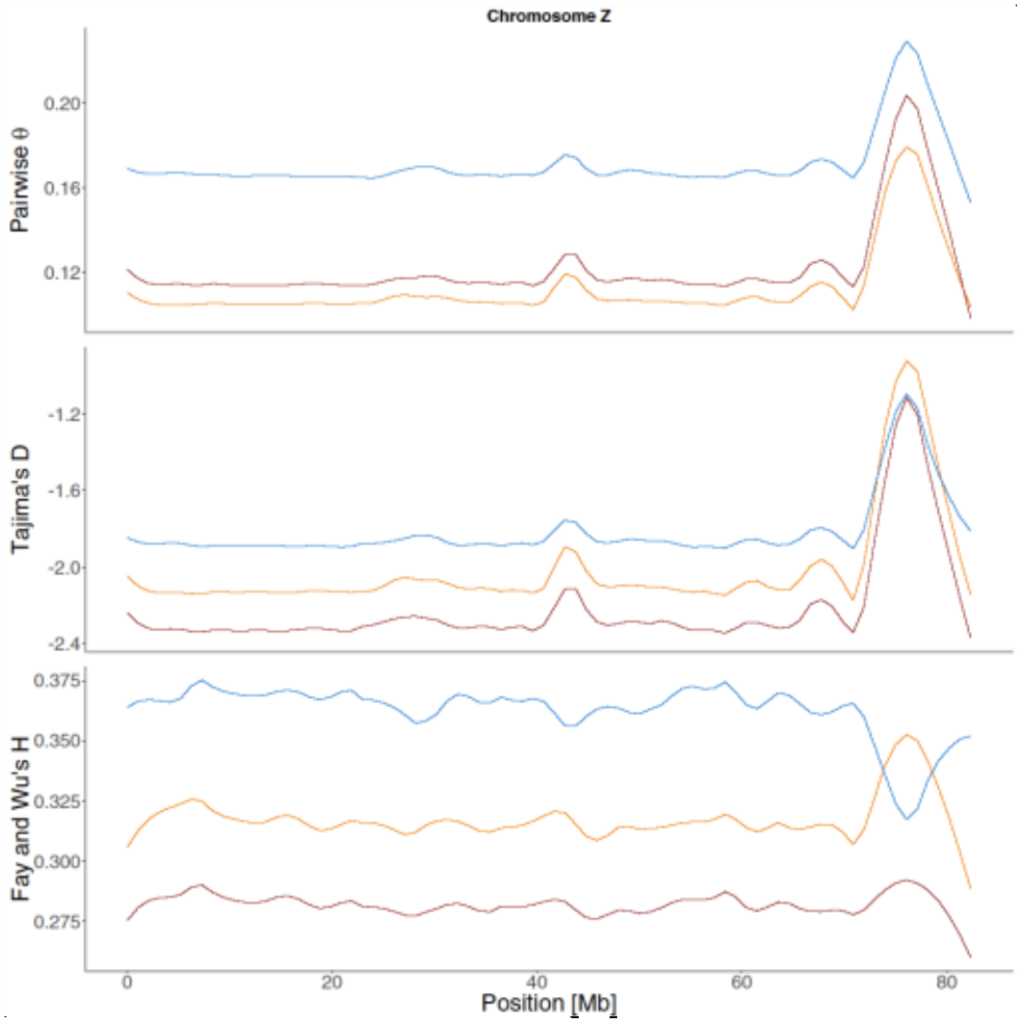

Supplement: Supplementary file 1 — Supplementary Information [file 41598_2018_36880_MOESM1_ESM.pdf]
